# Levodopa–Entacapone–Carbidopa Intestinal Gel in the Treatment of Advanced Parkinson’s Disease: A Single Center Real-World Experience

**DOI:** 10.3390/pharmaceutics16040453

**Published:** 2024-03-25

**Authors:** Szabolcs Szatmári, József Attila Szász, Károly Orbán-Kis, Beáta Baróti, Simona Bataga, Marius Ciorba, Előd Ernő Nagy, Radu Mircea Neagoe, István Mihály, Péter Zsombor Szász, Krisztina Kelemen, Attila Frigy, Mónika Szilveszter, Viorelia Adelina Constantin

**Affiliations:** 1 2nd Clinic of Neurology, Târgu Mures County Emergency Clinical Hospital, 540136 Târgu Mureș, Romania; szabolcs.szatmari@umfst.ro (S.S.); vioreliaconstantin@yahoo.com (V.A.C.); 2 George Emil Palade University of Medicine, Pharmacy, Science and Technology of Târgu Mures, 540142 Târgu Mureș, Romania; beatabaroti@yahoo.com (B.B.); simona.bataga@umfst.ro (S.B.); istvan.mihaly@umfst.ro (I.M.); peterszasz32@gmail.com (P.Z.S.); attila.frigy@umfst.ro (A.F.);; 3 Clinic of Radiology, Târgu Mures County Emergency Clinical Hospital, 540136 Târgu Mureș, Romania; 4 Department of Gastroenterology, Târgu Mures County Emergency Clinical Hospital, 540136 Târgu Mureș, Romania; 5 Laboratory of Medical Analysis, Clinical County Hospital Mures, 540072 Târgu Mureș, Romania; 6 2nd Clinic of Surgery, Târgu Mures County Emergency Clinical Hospital, 540136 Târgu Mureș, Romania; 7 Department of Neurology, Emergency County Hospital, 530173 Miercurea-Ciuc, Romania; 8 Department of Internal Medicine IV, Clinical County Hospital Mures, 540072 Târgu Mureș, Romania

**Keywords:** advanced Parkinson’s disease, levodopa–entacapone–carbidopa intestinal gel, motor fluctuations, peak-dose dyskinesia, diphasic dyskinesia, freezing

## Abstract

Levodopa–entacapone–carbidopa intestinal gel infusion is a relatively new treatment option for advanced Parkinson’s disease. We aimed to describe and analyze the characteristics of de novo levodopa–entacapone–carbidopa intestinal gel therapy in 20 consecutive patients with advanced Parkinson’s disease. We assessed the profile of motor complications by evaluating the following: motor fluctuations, dyskinesias, and the freezing phenomenon at baseline (before the testing period) and before discharge. The treatment significantly reduced the duration of daily hours spent in off time compared with baseline pre-treatment values from a mean of 4.8 ± 0.9 h/day to a mean of 1.4 ± 0.5 h per day (*p* < 0.001). The duration and severity of peak-dose dyskinesia were also significantly reduced compared with baseline values. Out of the 10 patients who reported freezing, 8 did not present this complication at the pre-discharge assessment. Significant improvements were observed in Hoehn and Yahr scale scores in both the on and off states. The levodopa–entacapone–carbidopa intestinal gel therapy was well tolerated during the follow-up period immediately after initiation. Despite a relatively severe stage of the disease, all patients experienced a significant improvement in motor fluctuations, dyskinesias, and the freezing phenomenon.

## 1. Introduction

Parkinson’s disease (PD) is considered a neurodegenerative disorder with symptoms that can be treated effectively, mainly by pharmacological correction of the reduced dopaminergic tonus [1]. Nevertheless, the major challenge is to ensure a stable postsynaptic dopamine level [2,3]. Dopaminergic replacement therapy with levodopa (LD) still remains the gold standard of symptomatic efficacy in antiparkinsonian therapy. However, as the disease progresses, the therapeutic efficacy of LD is gradually narrowed and becomes unpredictable, whereas the side effects become more frequent and bothersome. Although the different combinations of levodopa and peripheral dopa decarboxylase inhibitors (carbidopa or benserazide) preparations are long-term efficacious and, in general, well tolerated, their clinical utility over time is frequently limited by the development of troublesome motor complications (motor fluctuations and dyskinesias) [4]. The continuous enteral infusion of LD allows for the bypassing of problems related to irregular gastric emptying and the consequential unpredictable intestinal absorption of orally administered LD, which significantly affects its bioavailability and contributes to the development and persistence of different motor complications [5]. The use of a gel containing levodopa and carbidopa in this way has been used for several years and proved convincingly effective [6,7,8]. The usual treatment duration is typically 16 wake hours, and additional benefits (both motor and nonmotor symptoms improvement) of 24 h levodopa–carbidopa intestinal gel (LCIG) administration have been reported in several case series and small clinical studies [9,10,11,12].

Neurologists from selected centers in Romania have more than ten years of experience with device-aided therapies (DAT). However, the only realistically available DAT so far was the LCIG therapy, as the number of patients receiving other device-aided treatment was extremely low [13,14]. Starting in 2021, levodopa–entacapone–carbidopa intestinal gel (LECIG) has been approved in our country, and university centers may offer this treatment to patients with advanced Parkinson’s disease (APD) based on clear clinical indications.

Entacapone is a highly selective, potent, and specific peripheral catechol-O-methyltransferase inhibitor (COMT-i) [15,16]. Used widely as a first-line strategy for over two decades, entacapone prolongs the half-life of levodopa and increases its bioavailability. It has been shown to improve the clinical benefits of individual LD doses when given to PD patients with motor fluctuations (end-of-dose deterioration, wearing-off phenomenon) [17]. Levodopa–entacapone–carbidopa intestinal gel infusion is a relatively new device-aided treatment option. Due to the presence of entacapone, the bioavailability of levodopa from levodopa–entacapone–carbidopa intestinal gel infusion is 20–25% higher than that from levodopa–carbidopa intestinal gel infusion [18]. Furthermore, a study published in 2020 demonstrated that the simultaneous administration of entacapone to LCIG administration results in a 36.5% lower apparent levodopa clearance, and there is a need for lower continuous maintenance doses, regardless of patients’ catechol-O-methyltransferase genotype [19]. This triple combination allows for achieving the same stable and effective plasma LD levels with lower daily doses of levodopa [18].

## 2. Materials and Methods

In this retrospective analysis, between December 2021 and July 2023, we enrolled 20 consecutive patients with advanced Parkinson’s disease, naive to enteral levodopa infusion, repeatedly evaluated and initiated on levodopa–entacapone–carbidopa intestinal gel (LECIGON^®^, Britannia Pharmaceuticals Limited, Reading, UK) treatment in our university teaching hospital.

Demographic data and various clinical parameters were recorded for all patients: age, gender, disease duration, disease severity (in both the on and off phases; measured using the Hoehn and Yahr scale), the profile of motor complications, and Mini Mental State Examination (MMSE). The administered doses of levodopa (total daily dose and dosing frequency) and different add-on therapeutical options: dopamine agonists (DA), MAO-B inhibitors (MAO-Bi), COMT-i, and amantadine were evaluated at baseline and after starting LECIG treatment. According to national regulations, the patients were hospitalized for titration and initiation onto levodopa–entacapone–carbidopa intestinal gel, for adjustment of doses, and for assessment of the efficacy of treatment. Calculation of the estimated LECIG morning dose and continuous infusion rate was performed according to literature recommendations [20,21]. During the titration period (on naso-jejunal testing phase) and after insertion of the percutaneous endoscopic gastro-jejunostomy (PEG-J system), the levodopa–entacapone–carbidopa intestinal gel doses were continuously adjusted to achieve maximum therapeutic benefit.

We assessed the profile of motor complications by evaluating the following: motor fluctuations (duration of off periods, presence of early morning akinesia, delayed on respectively no on), dyskinesias (peak-dose, diphasic, end of dose dystonia) and the freezing phenomenon, at baseline (before the testing period) and on the day before discharge (after PEG-J and continuous dose adjustments). In our university teaching hospital, patients with advanced Parkinson’s disease underwent a two-step evaluation in order to assess the suitability for continuous enteral infusion of levodopa. During the first evaluation, after a detailed multidisciplinary evaluation (including a review of previous medical letters and discharging reports), and after proper preparation (if necessary, involving the caregivers, demonstration videos, etc.), the patient completes a diary (every 30 min) based on which at least one transition from on to off state (or vice versa) can be confirmed. Patients then received three 24-h logs that had to be completed three days prior to hospitalization. Due to national regulations, both the testing to demonstrate the effectiveness of the interstitial gel as well as the PEG procedure and the post-PEG adjustments were performed under continuous hospitalization conditions. In all cases of the present evaluation, the naso-jejunal tube was mounted passively without the need for radiological control. All patients were considered responders after testing.

Statistical analysis was performed using the Prism 8.0 software package (GraphPad Software, San Diego, CA, USA). The normality of data pools was tested each time. Depending on the type of data, descriptive statistics, parametric or non-parametric *t*-tests, and Kruskal–Wallis test were used. For contingency tables, Fisher’s exact test was performed. The level of statistical significance was *p* < 0.05 (non-significant values are marked as ns).

## 3. Results

We aimed to describe and analyze the characteristics of de novo levodopa–entacapone–carbidopa intestinal gel therapy in consecutive patients with advanced Parkinson’s disease, considered suitable for levodopa–entacapone–carbidopa intestinal gel. The demographic data and various clinical parameters are presented in Table 1.

The evaluation of concomitant medication usage at baseline shows that 80% of patients had dopamine agonists and COMT-i’s in their last dopaminergic treatment before LECIG initiation (two also with apomorphine as rescue medication). Furthermore, 70% received MAO-Bi treatment as well. After repeated adjustments during the titration phase after PEG-J, we managed to progressively reduce and subsequently stop dopamine agonists and MAO-Bi in five patients.

**Figure 1:**
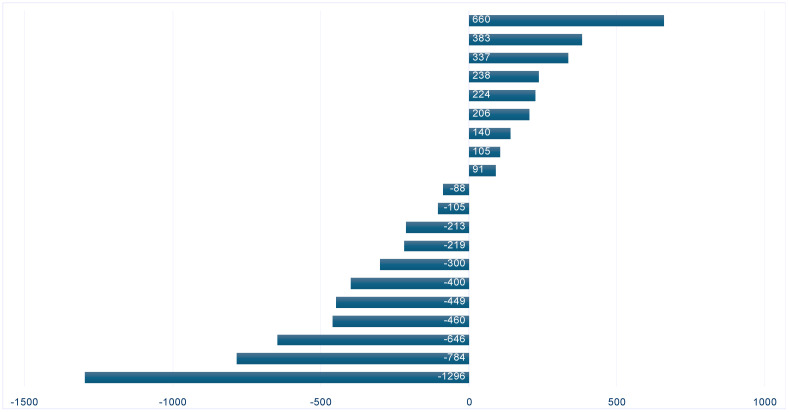
The differences between the theoretically calculated and real levodopa/entacapone/carbidopa intestinal gel doses. Negative values show lower actual LECIG doses compared to the theoretically calculated, whereas positive values present cases where the actual LECIG dose was higher.

Data on the duration of the test period on the naso-jejunal probe, post-PEG period, and until patient discharge (the fine-tuning of the LECIG doses to achieve the desired maximum therapeutic benefit) and the respective doses used (morning dose, continuous rate, and number of extra doses) are shown in Table 2. The duration of enteral infusion was 16 h in 10 cases; in 10 other cases, in order to obtain a significant improvement of the symptomatology, there was a need for an 18-hour infusion. In Figure 1, we presented the differences between the theoretically calculated (LEDD: 1330.2 ± 334.7 mg) and real levodopa/entacapone/carbidopa intestinal gel doses (1201.4 ± 273.8 mg), showing a decrease in doses in the case of 11 patients. The final evaluation was performed the day before discharge. During the evaluation period, no patient presented any significant complication; 16/20 patients reported minor discomfort/pain (post-procedural pain). No gastrointestinal adverse effects were reported until the time of discharge. Entacapone-naive patients were additionally evaluated; we did not find any adverse effects, and the laboratory results were not modified during pre-discharge testing.

Levodopa–entacapone–carbidopa intestinal gel treatment significantly reduced the duration of daily hours spent in off time compared with baseline pre-treatment values from a mean of 4.8 ± 0.9 h/day to a mean of 1.4 ± 0.5 h per day (*p* < 0.001). The duration and severity of peak-dose dyskinesia were also significantly reduced compared with baseline values (Table 3). None of the three patients with severe peak-dose dyskinesia had this type of complication at discharge. The relatively high number of patients suffering from end-of-dose dystonia showed significant improvement when the LECIG treatment started (*p* < 0.01). Early morning akinesia was documented in the majority of our APD patients; the improvement in their case was also significant (*p* < 0.001). Of the 10 patients who reported freezing, 8 did not present this complication at the pre-discharge assessment. Significant improvements (*p* < 0.01) were observed in Hoehn and Yahr scale scores in the off state after initiation of LECIG treatment, and sudden off episodes of the 4 patients disappeared too.

## 4. Discussion

Parkinson’s disease is the second most common neurodegenerative disorder and is thought to be caused by multiple pathomechanisms (mitochondrial dysfunction, aging, synuclein aggregation, neuroinflammation, and increased oxidative stress) [22,23]. Despite many proven symptomatic therapies successfully applied for PD, substitution therapy with levodopa formulations is key for the best clinical improvement at all stages of Parkinson’s disease [2]. A major disadvantage of long-term LD treatment is the occurrence of motor complications that significantly impair the quality of life [24,25]. Several ways of improving the unfavorable pharmacokinetics of levodopa have been widely used; additional positive effects can be expected from the introduction of the third-generation COMT inhibitor opicapone [26,27] and the MAO-B inhibitor and glutamate modulator safinamide [28,29]. However, both are still unavailable in Romania, as in most countries in Central and Eastern Europe.

Advanced Parkinson’s disease is characterized by motor fluctuation (periods of poor mobility), different dyskinesias (peak-dose, diphasic, dystonia), progressive decline in functional independence, and continuous worsening of quality of life (QoL). With an extremely heterogeneous and constantly changing clinical picture, the therapeutic strategy must be continuously adapted under conditions of strict personalization. This is also true for the use of different DAT options (enteral infusions of levodopa, subcutaneous infusion of apomorphine, or deep brain stimulation (DBS), which are expected to improve motor and non-motor complications and thus health-related QoL [30,31,32]. Non-oral LD delivery bypasses issues with gastrointestinal transport and absorption that may compromise oral treatment and has been developed along two main rationales: to provide stable plasma concentrations via continuous drug delivery and to provide a rapid onset of clinically beneficial effect suitable for use as an on-demand (“rescue”) medication. The LCIG delivered continuously into the upper intestine via a PEG-J, bypassing the very common gastroparesis in APD [33], provides more stable plasma levels compared to oral LD. Levodopa-carbidopa intestinal gel is a long-term proven and effective treatment in advanced Parkinson’s disease with severe motor fluctuations, with or without dyskinesias, and has also been shown to improve the nonmotor complaints commonly associated with chronic oral LD therapy [6,7,8]. Recent evidence suggests that LCIG significantly reduced dyskinesia compared with oral-optimized medical treatment [34]. The tolerability and safety profile of LCIG is generally comparable with that of oral LD therapies, with the exception of events related to the delivery system and its placement [35]. Furthermore, a recent systematic review and meta-analysis demonstrated that enteral infusion of levodopa-carbidopa intestinal gel has comparable efficacy to the deep brain stimulation of subthalamic nuclei on motor functions in APD patients, with acceptable tolerability [36].

Catechol-O-methyltransferase is an important enzyme involved in the peripheral and central metabolism of dopamine [16]. COMT-is are known as key therapeutic options in the long-term management of motor fluctuations. By blocking the second-largest degradation pathway and reducing the peripheral metabolization of levodopa, they allow higher amounts of levodopa to reach the brain (extend the duration of motor benefit from individual LD doses). As add-on therapy to standard levodopa formulations, their main benefit lies in increasing on-time and reducing off-time in the middle stages of Parkinson’s disease [1,18]. The widely available entacapone has been approved for over two decades. Entacapone is rapidly absorbed, with the plasma peak concentration occurring at approximately 1 h. The absorption is highly variable between different individuals and exhibits high intraindividual variability as well. It is poorly lipophilic and does not penetrate the blood-brain barrier (acting mainly in the gut its clinical effects are thus due to peripheral COMT inhibition only). Entacapone also slowed the elimination of LD from plasma [37]. The pharmacokinetics of entacapone appear to be linear, quite similar to those of levodopa, and, furthermore, entacapone is not influenced by food intake [17,38].

The combination of levodopa enteral infusion and the oral COMT-i (both entacapone and tolcapone) was tested previously, and the dose of LCIG could be decreased by 20% while maintaining stable levodopa plasma levels and motor function [39]. Levodopa–entacapone–carbidopa intestinal gel represents a novel treatment option for intrajejunal levodopa administration. Compared to levodopa–carbidopa intestinal gel, LECIG contains COMT inhibitor entacapone (20 mg/mL) in addition to levodopa (20 mg/mL) and carbidopa (5 mg/mL). Levodopa–entacapone–carbidopa intestinal gel is delivered using the Crono^®^ LECIG pump, which is smaller and lighter than the pump used to deliver LCIG. The presence of entacapone in LECIG increases the bioavailability of levodopa, which means that lower overall levodopa doses can be given while still achieving therapeutically effective plasma concentrations [21,40]. The effectiveness of levodopa–entacapone–carbidopa intestinal gel to motor symptoms appears similar to LCIG treatment observed in one 48 h crossover study [18]. The initiation of levodopa–entacapone–carbidopa intestinal gel requires the same surgical procedure as for LCIG infusion, namely the insertion of a PEG-J system so that medication can be delivered directly into the jejunum [21].

The first clinical experience with LECIG, based on the clinical use and patient-reported outcome, was published in 2021 [41]. To the best of our knowledge, this retrospective evaluation is one of the first publications of a single-center experience with de novo patients initiated on levodopa–entacapone–carbidopa intestinal gel. All patients in our cohort were transitioning from oral/transdermal treatments to LECIG, and so underwent PEG-J insertion. However, in clinical practice, patients who have been treated previously with LCIG and have an existing PEG-J system can transition to LECIG if needed, using a connection adapter. When switching from levodopa-carbidopa intestinal gel to LECIG, the results suggest that the continuous dose needs to be decreased by approximately 35% on a population level [19].

In our clinic, there is an important, more than one decade experience with enteral infusion of levodopa [42]. In previous publications, we detailed our procedure for the selection of eligible APD patients, the evaluation modalities of different motor complications severity, the challenges of the testing process, and establishing effectiveness, all under the specific conditions of clinical practice in our country [43,44,45]. Analyzing the results obtained by our patients, we evidenced both the efficacy and safety of long-term administration of LCIG therapy in patients with severe motor fluctuations and complex dyskinesias [46].

In general, initiating treatment with enteral LD infusion needs a multidisciplinary team consisting of a movement disorder specialist or neurologist with expertise in movement disorders, a specialist PD nurse, a gastroenterologist or surgeon (for PEG-J system placement), and, in some cases, an interventional radiologist, a psychiatrist or a cardiologist. The aim of this complex, multidisciplinary evaluation is to establish the suitability for DAT. Also, the testing of the efficiency of LD enteral infusion via a temporary naso-jejunal tube and the selection of the most convenient dosage regimen was performed in every case during continuous hospitalization [1,42]. Regarding the time of initiation, the length of the naso-jejunal testing phase and the management of the previous add-on dopaminergic medication were largely left to the clinician’s personal options and experience. There is a lack of clear recommendations from experts, although these would be particularly important for increasing the long-term safety and efficacy and lowering the discontinuation rate. In the last years, there have been published expert opinions that the initiation of enteral infusion of LD can be initiated with the help of telemedicine, under outpatient titration or even without the NJ testing phase [47,48]. Due to the potential of overlooking factors that would increase the dropout rate, we believe that these options should be used only in cases where access to a tertiary center and implicitly the possibility of regular check-ups or the resolution of complications in an emergency regime is limited (e.g., large distances, already institutionalized patients, limitation of hospitalizations due to unforeseen circumstances, such as pandemics, etc.) [44]. However, indubitably, the naso-jejunal tube may cause discomfort to the patient, is time-consuming, and is susceptible to complications.

It is known that there are no unanimously accepted criteria for the identification of advanced Parkinson’s disease, nor for the optimal time for initiating device-aided therapy (considering predictable efficacy and long-term safety) [1,43]. In the second half of 2015, an expert statement was published stating that if a patient with advanced Parkinson’s disease develops 2 h of off-state and/or 1 h of severe dyskinesia per day despite five times daily doses of combined LD therapy (“optimized medication”), DAT options should be considered [30]. This “5-2-1 rule” has been further refined in subsequent publications [49,50,51,52].

In daily clinical practice, patients treated with LD enteral infusion have a more severe clinical picture (both in studies found in the literature and in our own work) compared to expert recommendations [45,53,54]. This trend is also observed in the profile of motor complication in our patients treated with levodopa–entacapone–carbidopa intestinal gel: mean cumulative off duration 4.8 ± 0.9 h and in 65% of cases peak dose dyskinesia (3.0 ± 1.3 h). In this context, we also note the increased incidence of early morning akinesia (90% of cases), 50% of which showed delayed and the freezing phenomenon was also observed in 50% of cases (Table 2).

Previous analysis suggests as a characteristic of APD management in our region, more frequent usage of different add-on therapeutic options (DA’s, MAO-Bi’s, and entacapone as the only one available COMT-i) prior to initiation of enteral infusion of LD [42]. Similarly, in terms of the last dopaminergic treatment previously to the evaluation for eligibility for LECIG, the proportion of dopamine agonists (80%) and the MAO-Bi’s (70%) was found to be significantly higher compared to other literature reports. A particular situation in this context is the use of entacapone as the only available add-on option from the COMT-i class (used in 80% of patients prior to LECIG initiation, a proportion clearly higher than the literature data) [42]. One adverse effect of entacapone that needs to be considered is diarrhea. Experts recommend that if the patient has been previously treated without adverse effects, levodopa–entacapone–carbidopa intestinal gel can be initiated directly [40,55]. An earlier study has shown that patients who suffered from diarrhea with oral entacapone also developed it during LECIG treatment [41]. If no data are available, an oral entacapone trial prior to testing is recommended. As the experience using LECIG was limited when it became available in our country, we selected for initiation mostly patients who previously also had entacapone therapy. In a recently published retrospective evaluation of 74 APD patients treated with LECIG at 12 specialized APD centers in Romania, we showed that during the titration period as well as the post-PEG adjustment period, we did not encounter any significant adverse effects compared to those mentioned in the literature regarding the PEG insertion procedure [56]. We would like to point out that in the present evaluation, none of the entacapone-naive patients experienced gastrointestinal adverse effects; furthermore, none of the patients showed modified laboratory results on further testing. We consider that it is still premature to comment on the clinical criteria, which can help the decision of choice for any of the enteral infusion solutions of levodopa, but the previous improvement of off episodes with entacapone may represent a possible argument in favor of therapy with levodopa–entacapone–carbidopa intestinal gel.

The presence of entacapone in LECIG increases the bioavailability of LD (lower overall LD doses can be given while still achieving clinically effective plasma concentrations). We used levodopa–entacapone–carbidopa intestinal gel doses at initiation, similar to published studies, with the mention that our patients were discharged without other added levodopa treatment [41,57]. The short-term beneficial effects were also similar to these studies; however, our patients did not have immediate complications (i.e., in the first days after the insertion of PEG-J) except for a minor local discomfort/pain.

Using the lowest possible clinically effective dose of levodopa has potential long-term benefits. Peripheral neuropathy is frequently reported in Parkinson’s patients receiving prolonged treatment with oral or intestinal infusion of LD, with a significantly higher prevalence of acute and subacute forms in those receiving LCIG [58,59]. The development of peripheral neuropathy has been linked to the duration and dose of levodopa and also to high levels of the levodopa metabolite, homocysteine [60]. Entacapone, which blocks the methylation pathway of levodopa metabolism that results in the generation of homocysteine, has been shown to have a protective effect against the development of peripheral neuropathy [61]. In a single-center retrospective study of 30 APD patients treated with levodopa–entacapone–carbidopa intestinal gel (de novo and switched from LCIG) published in 2023, no clinically diagnosed cases of polyneuropathy appeared during the first six months of levodopa–entacapone–carbidopa intestinal gel treatment. Furthermore, no increase was seen in homocysteine levels [57].

Another very important practical aspect of APD management is the decrease in patients’ therapeutic adherence following sophisticated therapeutic formulations [62]. The high pill burden secondary to complex treatment strategies represents an important factor that worsens both patients’ quality of life and compliance [63,64]. In the analyzed group, in five cases after PEG-J, we succeeded in discontinuing dopamine agonists and rasagiline, respectively. In the other cases, we progressively reduced the dose of dopamine agonists (previous experience has shown that the post-PEG-J titration and adjustment period, even in conditions of continuous hospitalization, is not sufficient for the discontinuation of dopamine agonists) and then, depending on the subsequent evolution, we will discontinue it if possible.

The treatment of Parkinson’s patients in our center follows international guidelines, with a similar strategy to that followed by specialist teams in other countries [65,66,67]. Therefore, we believe that this work may be useful especially for clinicians in Central and Eastern Europe who are caring for PD patients with similar therapeutic possibilities. In order to maximize the long-term benefits of different device-aided therapies is very important to realize without delay that the limits of conventional medication have been reached, and in this stage of advanced Parkinson’s disease, it is imperative to refer the patient to a movement disorders center with multidisciplinary teams with expertise in initiating accessible DATs.

## 5. Limitations of the Study

The strengths of this analysis are presented by the relatively large number of APD patients from real-life clinical practice, naive to the intrajejunal levodopa infusion initiated on this new therapeutical option. However, this study has several limitations as well. Firstly, we are aware of the fact that the cross-sectional analysis of the initiation period is a notable limitation as it does not allow us to make additional clarifications regarding the longer-term evolution and the safety of the long-term therapy. Furthermore, studies are needed to (i) more clearly define the category of patients who could benefit the most from the treatment with levodopa–entacapone–carbidopa intestinal gel, (ii) gather additional data on long-term efficacy, and (iii) analyze the reduction of combination medication over time. Further collection of clinical data via registries will provide valuable information to support clinical practice observations and clinical trial evidence. One such study is the ELEGANCE study (NCT05043103), the Global Long-Term Registry on Efficacy and Safety of LEciGon in Patients with Advanced ParkinsoN’s Disease in Routine CarE, which is a non-interventional observational study that aims to provide additional long-term real-world data on the efficacy and safety of LECIG used in the clinical practice setting at centers in around 16 countries in Europe [68].

## 6. Conclusions

Intrajejunal infusion of levodopa is a long-time proven useful treatment option for advanced Parkinson’s disease. The emergence of a new formula that includes entacapone allows for obtaining comparable clinical benefits with lower total daily doses of levodopa.

In our group of patients with advanced Parkinson’s disease, the treatment with levodopa–entacapone–carbidopa intestinal gel was well tolerated during the follow-up period immediately after initiation. Despite a relatively severe stage of Parkinson’s disease, when device-aided therapy was accepted, all patients experienced a significant improvement in motor fluctuations, dyskinesias, and the freezing phenomenon. As more clinical experience with the therapy with levodopa–entacapone–carbidopa intestinal gel accumulates one can assume the profile of the patient who will benefit to the maximum from these device-aided treatment alternatives can be defined much more clearly.

## Figures and Tables

**Table 1:** Baseline characteristics of APD patients treated with LECIG.

Characteristics	Baseline Data (*n* = 20)
Male, *n* (%)	10 (50%)
Female, *n* (%)	10 (50%)
Age, years (mean ± SD), all patients min max	65.8 ± 7.1 50 79
Time since PD diagnosis, years (mean ± SD)	10.2 ± 4.5
Hoehn-Yahr scale score (mean ± SD) On state Off state	3.25 ± 0.4 4.15 ± 0.4
Mini Mental State Evaluation score (mean ± SD)	27.4 ± 1.8
Levodopa daily dose, mg (mean ± SD) min max	864 ± 291 500 1750
Levodopa dose frequency, number per day (mean ± SD)	5.4 ± 0.6
Dopamine agonists, *n* (%)	16 (80%)
MAO-B inhibitors, *n* (%)	14 (70%)
COMT inhibitors, *n* (%)	16 (80%)
Amantadine, *n* (%)	5 (25%)

**Table 2:** Characteristics of testing and adjusting LECIG in APD patients.

Najo-jejunal testing phase (days, mean ± SD)	5.55 ± 1.1
Post-PEG adjustment (days, mean ± SD)	4.2 ± 0.5
LEDD (mg, mean ± SD)	1330.2 ± 334.7
LECIG real (mg, mean ± SD)	1201.4 ± 273.8
LECIG administration Morning dose (ml, mean ± SD) Continuous rate (ml, mean ± SD) Extra dose (ml, mean ± SD)	5.6 ± 1.4 2.9 ± 0.7 1.6 ± 0.6
Administration time 16 h (*n*) 18 h (*n*)	10 10

**Table 3:** Changes in motor complications from baseline to the finishing of the adjustment of LECIG treatment.

Characteristic	Baseline (*n* = 20)	After LECIG Treatment (*n* = 20)	*p*-Value
Off time duration, hours per day (mean ± SD)	4.8 ± 0.9	1.4 ± 0.5	<0.001
Peak-dose dyskinesia *n* (%)	13 (65%)	11 (55%)	>0.05
Duration, hours per day (mean ± SD)	3.3± 1.3	1.2 ± 0.6	<0.001
Mild/moderate peak-dose dyskinesia, *n* (%)	13 (65%)	11 (55%)	
Severe peak-dose dyskinesia, *n* (%)	3 (15%)	0 (0%)	N/A
Diphasic dyskinesia occurrence, *n* (%)	1 (5%)	1 (5%)	N/A
End of dose dystonia occurrence, *n* (%)	8 (40%)	1 (5%)	<0.01
Early morning akinesia occurrence, *n* (%)	18 (90%)	4 (20%)	<0.001
Delayed ON occurrence, *n* (%)	10 (50%)	N/A	N/A
No ON occurrence, *n* (%)	3 (15%)	N/A	N/A
Sudden OFF occurrence, *n* (%)	4 (20%)	0(0%)	<0.05
Freezing occurrence, *n* (%)	10 (50%)	2 (10%)	<0.01
Hoehn and Yahr Scale scores:			
On state	3.25 ± 0.4	3.1 ± 0.3	ns
Off state	4.15 ± 0.4	3.45 ± 0.5	<0.001

## Data Availability

The data presented in this study are available on request from the corresponding author. The data are not publicly available due to privacy reasons.
